# The Role of Paracrine and Autocrine Signaling in the Early Phase of Adipogenic Differentiation of Adipose-derived Stem Cells

**DOI:** 10.1371/journal.pone.0063638

**Published:** 2013-05-28

**Authors:** Mette Hemmingsen, Søren Vedel, Peder Skafte-Pedersen, David Sabourin, Philippe Collas, Henrik Bruus, Martin Dufva

**Affiliations:** 1 Department of Micro- and Nanotechnology, Technical University of Denmark, Kgs. Lyngby, Denmark; 2 Institute of Basic Medical Sciences, Stem Cell Epigenetics Laboratory, Faculty of Medicine, University of Oslo, Oslo, Norway; 3 Department of Physics, Technical University of Denmark, Kgs. Lyngby, Denmark; University of California San Diego, United States of America

## Abstract

**Introduction:**

High cell density is known to enhance adipogenic differentiation of mesenchymal stem cells, suggesting secretion of signaling factors or cell-contact-mediated signaling. By employing microfluidic biochip technology, we have been able to separate these two processes and study the secretion pathways.

**Methods and results:**

Adipogenic differentiation of human adipose-derived stem cells (ASCs) cultured in a microfluidic system was investigated under perfusion conditions with an adipogenic medium or an adipogenic medium supplemented with supernatant from differentiating ASCs (conditioned medium). Conditioned medium increased adipogenic differentiation compared to adipogenic medium with respect to accumulation of lipid-filled vacuoles and gene expression of key adipogenic markers (C/EBPα, C/EBPβ, C/EBPδ, PPARγ, LPL and adiponectin). The positive effects of conditioned medium were observed early in the differentiation process.

**Conclusions:**

Using different cell densities and microfluidic perfusion cell cultures to suppress the effects of cell-released factors, we have demonstrated the significant role played by auto- or paracrine signaling in adipocyte differentiation. The cell-released factor(s) were shown to act in the recruitment phase of the differentiation process.

## Introduction

Adipose tissue regulates energy homeostasis and also works as an endocrine organ secreting many adipokines which regulate e.g. insulin sensitivity, immune function and lipid metabolism [1]–[3]. The tissue is composed of cells of mesodermal origin that are able to accumulate large amounts of triglycerides in cytoplasmic vacuoles. In addition to mature adipocytes, adipose tissue also contains multipotent stromal cells called adipose-derived stem cells (ASCs) [4]. ASCs have received much attention due to their high availability, the absence of ethical considerations regarding their obtention, and their potential in regenerative medicine [5], [6].

The differentiation of mesenchymal stem cells (MSCs, including ASCs) into adipocytes is divided into two steps [1]. The first step is a commitment to the adipogenic cell lineage by differentiation into preadipocytes which are morphologically indistinguishable from their precursor cells, but are limited in their differentiation capacity to only adipocytes [3]. In the second step, preadipocytes enter terminal differentiation to become functional adipocytes upon exposure to adipogenic stimuli. MSCs and preadipocytes proceed through adipogenic differentiation *in vitro* when cultured with a cocktail of adipogenic chemical stimuli such as dexamethasone, isobutyl-methylxanthine (IBMX), insulin and in some protocols indomethacin [4], [7], [8]. Human preadipocytes enter the differentiation program without cell division, while the mouse preadipocytes (e.g. 3T3-L1 cells) divide once or twice before differentiation [3].

Many molecular cues have been shown to be involved in regulation of adipogenesis [1]–[3]. However, two important groups are members of the transforming growth factor beta (TGFβ) superfamily [9] and the wingless-type mouse mammary tumor virus (MMTV) integration site family members (WNT) signaling molecules [10], [11], which are secreted glycoproteins operating in an auto/paracrine manner in many developmental processes. Treatment with the TGFβ superfamily member bone morphogenic protein 4 (BMP4), both prior and throughout differentiation, promotes adipogenesis in human ASCs [12] and human Simpson-Golabi-Behmel syndrome (SGBS) preadipocytes [13], whereas treatment only before induction of differentiation does not support adipogenesis in SGBS preadipocytes [13]. In contrast, BMP4 pretreatment of mouse pluripotent C3H10T1/2 cells increases adipogenic differentiation substantially [14], [15]. Conversely to the proadipogenic effect of BMP4 at high doses (50–100 ng/mL) [12]–[15], low doses of BMP4 (0.01-0.1 ng/mL) maintain stemness and self-renewal properties of human ASCs [16]. The role of TGFβ (the canonical member of the TGFβ superfamily) is unclear [1]. TGFβ inhibits adipogenesis in mouse preadipocytes [17]–[19], while increased TGFβ expression correlates with obesity in humans and mice [9], [20].

Of the WNT signaling molecules, WNT5A inhibits adipogenesis in human MSCs [21], while WNT6, WNT10A and WNT10B hinder adipogenesis in mouse preadipocytes by suppressing expression of CCAAT-enhancer-binding protein alpha (C/EBPα) and peroxisome proliferator-activated receptor gamma (PPARγ) [22], [23]. Furthermore, human adipocyte differentiation is associated with secretion of the WNT signaling inhibitors secreted frizzled-related proteins (sFRP) and Dickkopf-1 (Dkk1) [11], [24], which both hamper WNT signaling and thereby promote adipogenesis in human ASCs [12], [24]. Thus, WNT signaling may be an important regulator of adipocyte differentiation through a cross-talk between mature adipocytes and ASCs or preadipocytes, which further may be regulated by energy storage demands [10].

A transcriptional cascade is activated upon addition of adipogenic medium to MSCs and preadipocytes which results in terminal adipogenic differentiation and ultimately in expression of adipokines and adipocyte-specific metabolic proteins (such as leptin, adiponectin, lipoprotein lipase (LPL), fatty acid binding protein 4 (FABP4) and glucose transporter type 4 (GLUT4)) [1], [3], [25]. Many transcription factors have been shown to be involved in the terminal adipogenesis process; however, by far the most important and central transcription factors are PPARγ, C/EBPα, C/EBPβ and C/EBPδ [1], [26].

Although a number of previously reported findings suggest a possible role of auto/paracrine signaling in the differentiation process, the existence of such signaling within a population of MSCs is not well delineated [1], [3], [10], [25], [27]–[31]. Co-culture of human ASCs with human adipocytes enhances adipogenesis in adipogenic medium [32], and culture of mouse bone marrow MSCs in conditioned medium collected from mouse adipose tissue culture induces adipogenesis without any added chemical adipogenic factors [33]. Moreover, high cell confluence (80–90%) has been reported to be required to achieve efficient differentiation to adipocytes by adipogenic stimuli [30], [31], [34], [35]. Furthermore, high cell confluency has also been shown to promote adipogenesis over osteogenesis of human MSCs in a mixed adipogenic/osteogenic medium [36]. Finally, it is well known that differentiation in batch cultures is more efficient when exchanging only half of the adipogenic differentiation medium during replacement, which could be caused by cell-secreted signaling molecules retained in the non-replaced medium.

In this paper we investigate the possible existence of a critical auto/paracrine signaling pathway involved in the differentiation of human ASCs into adipocytes using microfluidic perfusion cultures for experimental observations and physical models for theoretical analysis of the associated advection-diffusion-reaction processes. Contrary to batch culture, microfluidics enables control of the cellular environment during differentiation [37], [38] since the media composition remains constant over the entire experiment. Time-resolved effects of the auto/paracrine network on adipogenic differentiation were determined by a set of complimentary measures consisting of the fraction of cells undergoing differentiation (recruitment), the degree of fat accumulation (functional test of adipogenesis) and finally the expression of molecular adipogenic markers (C/EBPα, C/EBPβ, C/EBPδ, PPARγ, LPL and adiponectin). Our results indicate that auto/paracrine signaling in a population of human ASCs exposed to adipogenic medium is an essential signal in the early phase of adipogenic differentiation.

## Results

### Numerical simulations of secreted factor accumulation in the chambers

**Figure 1:**
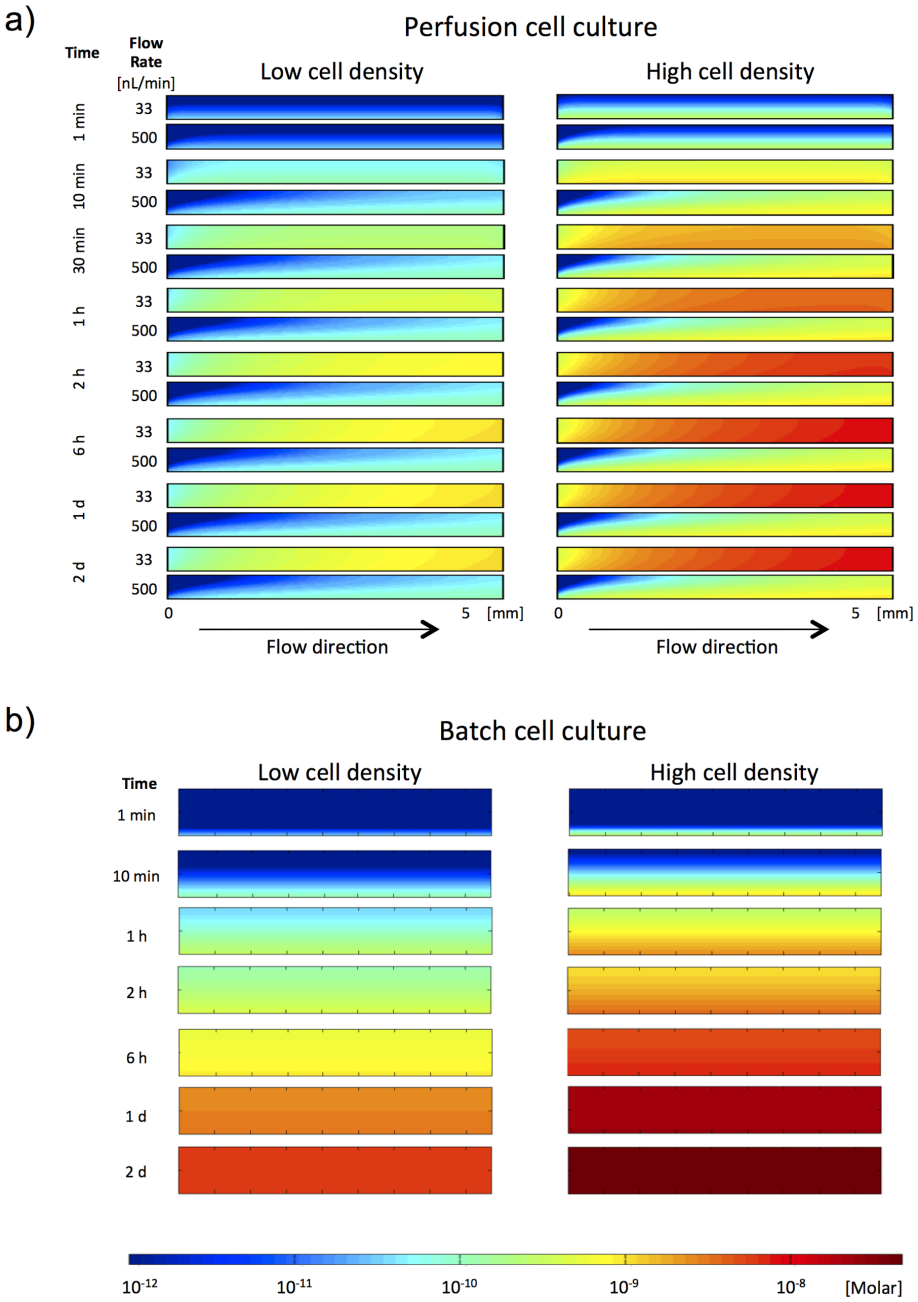
Numerical simulations of secreted factor accumulation in the chambers. Simulated distribution of a cell-released factor from cells residing at the bottom of A) a microfluidic chamber (*h* = 0.5 mm, *l* = 6 mm, *w* = 1.5 mm) perfused at 33nL/min or at 500 nL/min, and B) a conventional well (*d* = 17 mm and a height of medium of 2 mm) at either low cell density (left) or high cell density (right). The high cell density condition corresponds to cells covering the entire surface, while the low cell density condition was set 8 times lower than the high cell density. The rate of secreted factor was set to 100 molecules per cell per second, which is one order of magnitude lower than estimated antibody production in hybridoma cells [48], [49]. Simulation of released factor is shown from 1 second to 2 days. See Materials and Methods for more details about the analysis.

We wanted to investigate if ASCs or maturating adipocytes secrete a factor that enhances differentiation using a recently described parallel microfluidic system (Figure S1) to perfuse the cell cultures and thus wash out a secreted factor [39], [40]. A putative secreted factor would be subject to the combined effects of advection by the perfusion flow and diffusion in the microfluidic chamber. The distributions of the released factor in the perfused chambers were therefore investigated by solving numerically the governing equation of a soluble chemical at two cell densities and two flow rates with the relevant secretion and transport parameters (see Materials and Methods) (Figure 1). The model simulations showed that an equilibrium of released and washed out secreted factor was established after about 2 h in the case of perfusing at 33 nL/min and after 10 minutes by perfusion at 500 nL/min (Figure 1a). By contrast, the batch culture changed over time (Figure 1b). The model simulations also showed that high levels of a secreted factor are present in the case of high cell density in the low flow rate (33 nL/min) approaching the concentration in batch cultures (Figure 1). Moreover, figure 1a illustrates, that higher cell density in the chamber leads to higher factor concentration near the cells (variation of about one order of magnitude depending on cell concentration). Although the chamber volume is on average exchanged every 10 min at 500 nL/min, the medium close to the cells is exchanged about five times slower, resulting in significant build-up of factor concentrations in the medium at high cell densities. This is a result of the parabolically varying flow speed across the channel height with zero velocity at the walls and maximum velocity at the center.

It is, however, possible that low cell density and high perfusion rate will enable a null condition where cells cannot significantly modulate their own environment. This provides opportunities to study effects of the cell-released factor(s) in a minimal background.

### Effects of conditioned medium on differentiation

**Figure 2:**
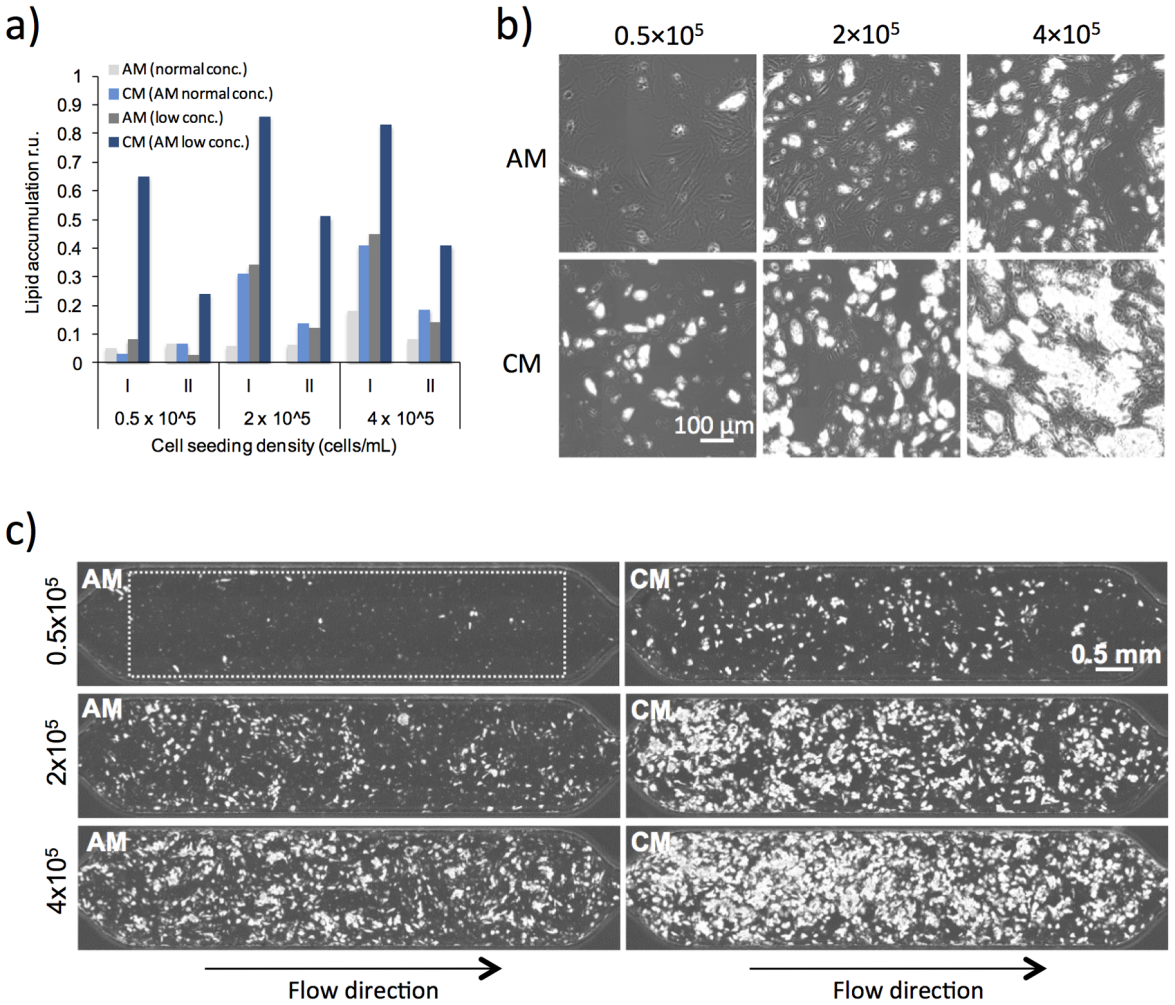
Effect of conditioned medium on adipogenic differentiation. ASCs were loaded at different cell suspension densities, 0.5×10^5^, 2×10^5^ or 4×10^5^ cells/mL, and induced to differentiate at a flow rate of 500 nL/min in AM or CM at two different concentrations of serum and adipogenic factors. CM was a 1∶1 mixture of supernatant from ASCs undergoing differentiation in batch cultures and fresh AM to ensure sufficient supply of nutrients. 1.5×AM was used in the mixture instead of 1×AM to compensate for an expected consumption/degradation of the adipogenic stimuli in the collected supernatant. A) Relative lipid accumulation in relative units. Lipid accumulation in the chambers are taken as a measurement of extent of differentiation, and was determined by quantifying the total area of lipid-filled droplets in each chamber (within the rectangle indicated by the dotted line in figure 2c) divided by the corresponding total cell area at the start of differentiation, see also material and methods. I and II denote two independent experiments. AM (low conc.) and CM (AM low conc.) indicate AM with 4 times lower concentrations of serum and adipogenic inducers and CM based on the 4 times lower concentrated AM. B) 10x phase contrast images 14 days after differentiation initiation of a representative area of a chamber at the different tested conditions. C) Scan of an entire cell culture chamber. The rectangle indicated by the dotted line shows the area of a cell culture chamber used for measurements of extent of differentiation with respect to total lipid area, lipid vacuole area per cell and fraction of differentiated cells (Figure 3 and Figure S3).

**Figure 3:**
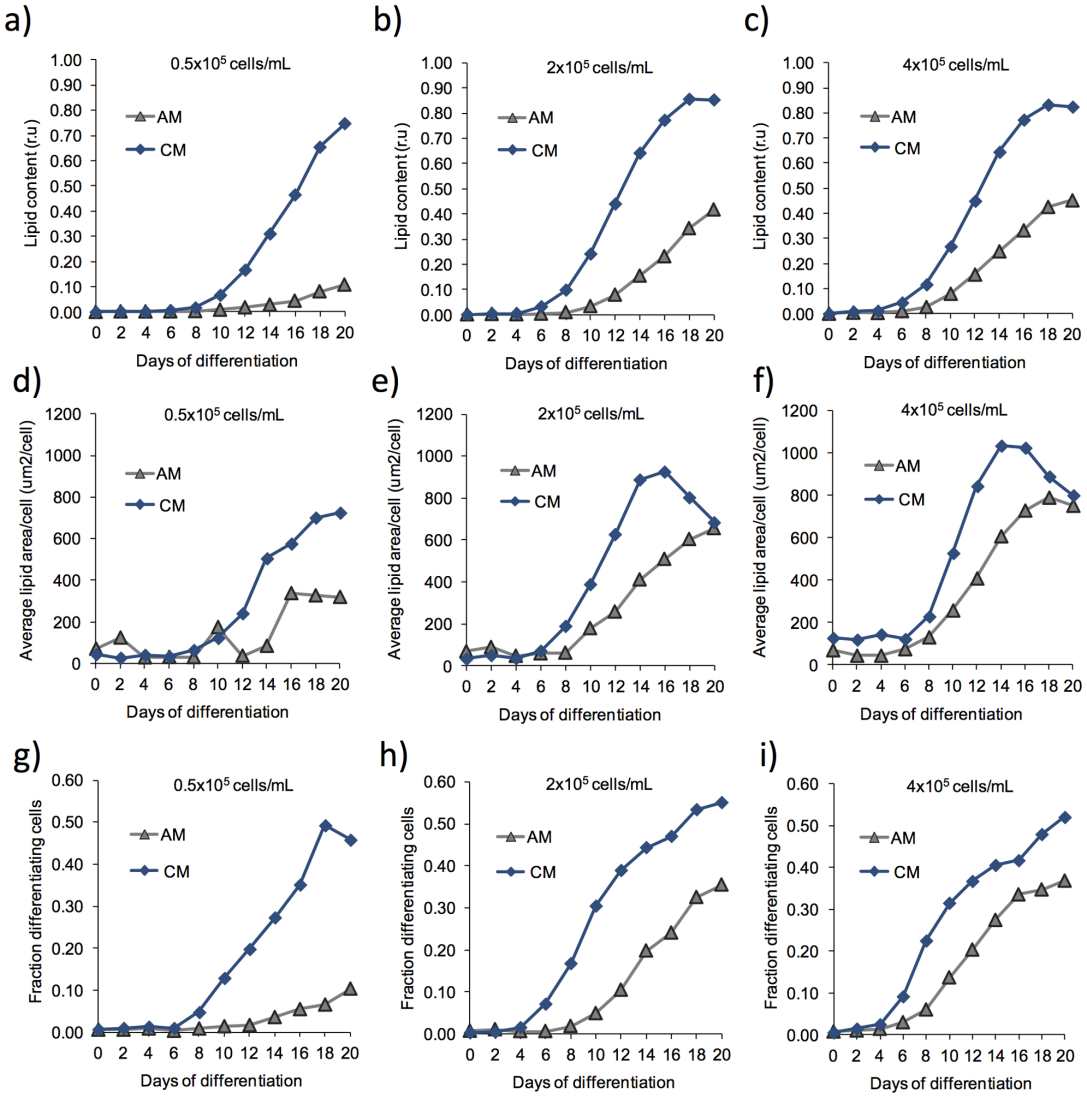
Time course study of lipid accumulation during adipogenic differentiation. Experiment I out of two independent experiments was followed over time. An image of cells in the entire cell culture chamber was captured every second day and relative lipid accumulation in relative units (A–C), lipid area per cell (D–F) and fraction of differentiated cells (G–I) was determined as described in material and methods. Each graph in the diagrams corresponds to analysis of one chamber. Corresponding results from experiment II are shown in Figure S3.

In order to suppress differentiation completely and to restore it with the putative secreted factor, a dilution series of cell densities were plated in the micro chambers and perfused at 500 nL/min with either adipogenic medium (AM) or conditioned medium (CM). Perfusion with AM provides cells with adipogenic stimuli while removing to large extent cell-released factors. Perfusion with CM provides cells with adipogenic stimuli but also cell released factors. As culture conditions are different between batch cultures and perfusion cultures as indicated by figure 1, we also tested two different concentrations of adipogenic medium. The ability to differentiate was monitored by intracellular lipid accumulation (Figure 2–3 and Figure S3). At all cell densities, CM had a positive effect (up to 9-fold) on differentiation compared to AM both with respect to fraction of differentiated cells and lipid accumulation (Figure 2–3 and Figure S3). The exception was differentiation at low cell densities with normal concentrations of adipogenic medium [7], [8] where no positive effect of CM was observed (Figure 2a). In fact, there was almost no differentiation in low density cell cultures perfused with AM while the ability to differentiate was restored by perfusing with CM (based on low concentration AM). By contrast, perfusion with an old batch of CM (supernatant collected from differentiating ASCs stored for 1–4 months at −20°C) did not result in more differentiation than perfusion with AM (data not shown), suggesting degradation or inactivation of the active components.

**Figure 4:**
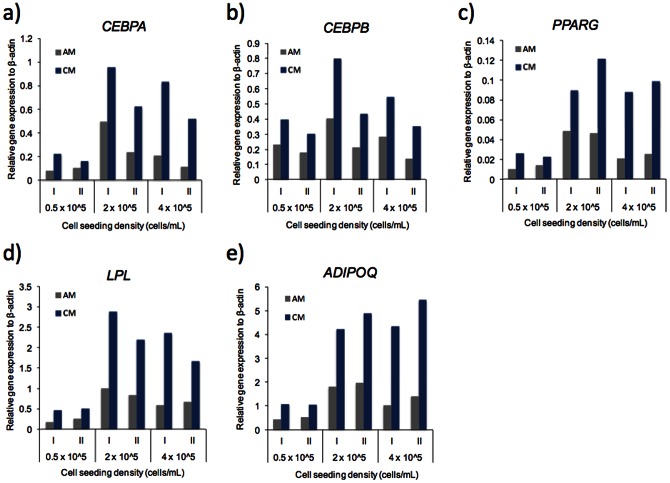
Quantitative measurements of gene expression of adipogenic markers. ASCs were loaded at different cell suspension densities, 0.5×10^5^, 2×10^5^ or 4×10^5^ cells/mL, and induced to differentiate at a flow rate of 500 nL/min in AM or CM. A–E) Gene expression of the adipogenic markers CEBPA, CEBPB, PPARG, LPL, and ADIPOQ was determined by reverse transcription real time PCR and normalized to expression of ACTB, see material and methods. (I) denotes experiment I and (II) denotes experiment II of two independent experiments.

**Figure 5:**
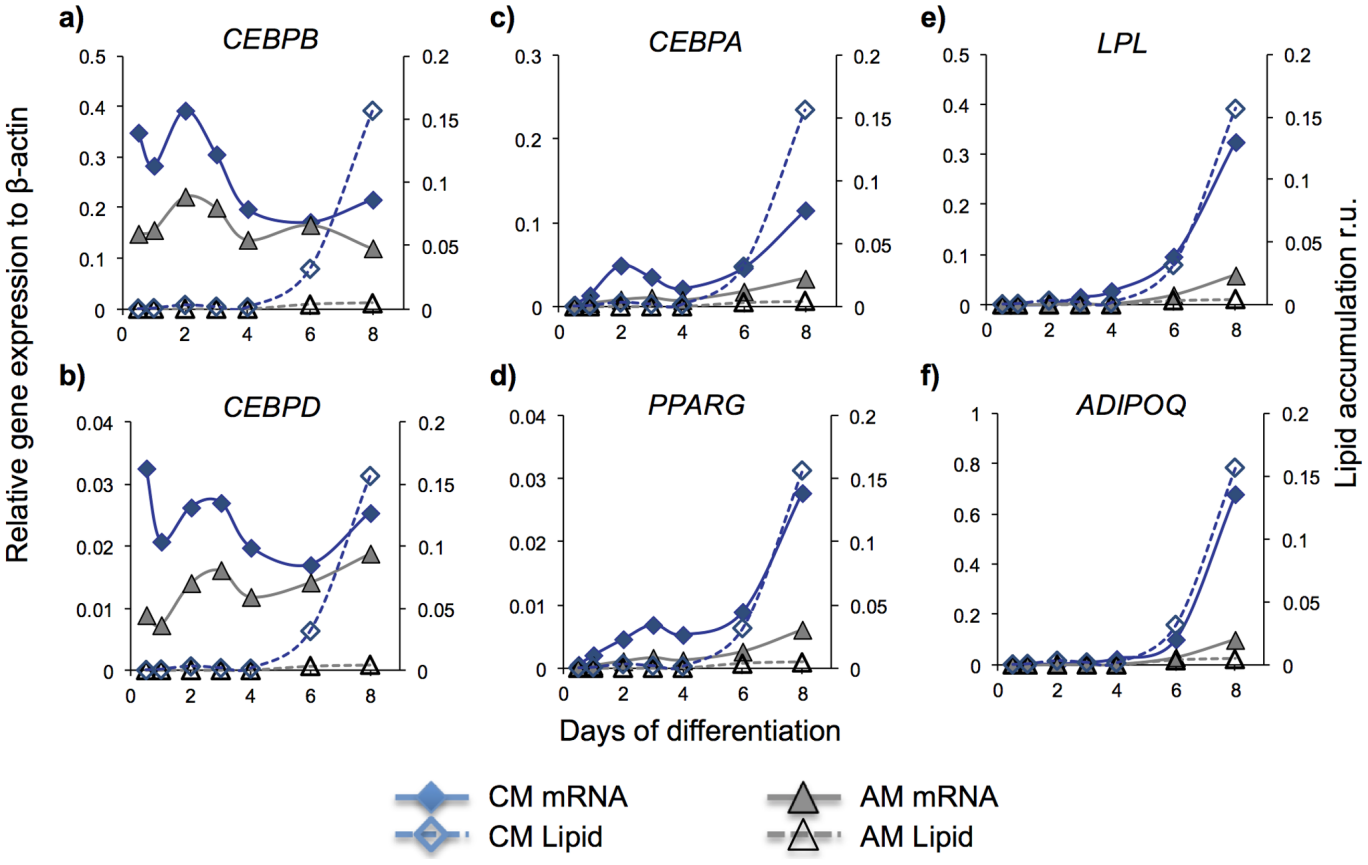
Effect of conditioned medium on gene expression of adipogenic markers early in the differentiation process. In an additional set of experiments ASCs were loaded at a cell suspension density of 2×10^5^ cells/mL and induced to differentiate at a flow rate of 500 nL/min in AM or CM. Gene expression of the adipogenic markers *CEBPB, CEBPD, CEBPA, PPARG, LPL* and *ADIPOQ* was analyzed by reverse transcription real time PCR of all cells in one cell culture chamber after 12 hours, 1, 2, 3, 4, 6, and 8 days of differentiation. The results shown are from experiment I out of three independent experiments. Results from experiments II and III are shown in Figure S4 and S5. Relative gene expression to β-actin shown on the left y-axis for A) *CEBPB*, B) *CEBPD*, C) *CEBPA*, D) *PPARG*, E) *LPL* and F) *ADIPOQ*. The corresponding lipid accumulation is shown on the right y-axis. Note the y-axis scales denoting the real-time PCR results are different in order to clearly visualize effects of CM on all adipogenic markers. Each point on the graphs corresponds to analysis of cells from one chamber.

**Figure 6:**
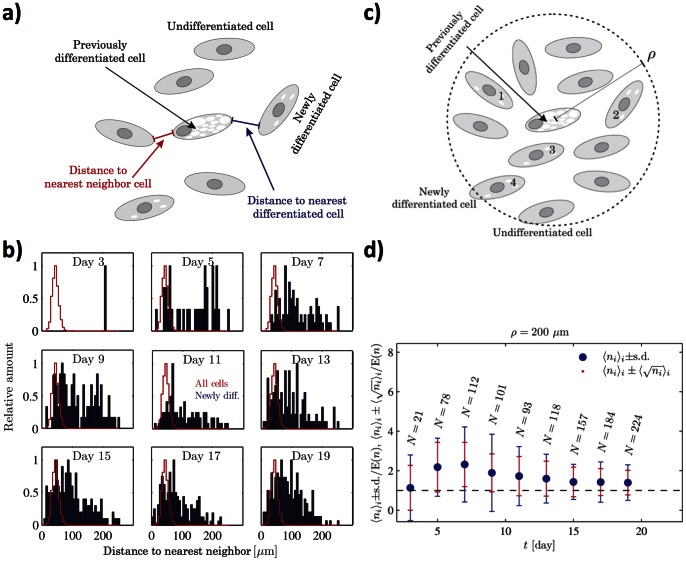
Source of factor secretion. A) Schematic illustration of the analysis used to investigate the potential increased differentiation due to positional proximity to already differentiated cells. B) Distributions of the average distances between newly differentiated cells and the closest already differentiated cell (blue) compared to the average distance between all nearest neighbor cells (red) for data at the cell density 270 cells/mm^2^ exposed to AM at Q = 500 nL min^−1^. Each plot in each panel is normalized by its largest value. C) Schematic illustration of the second independent analysis used to for investigating number of differentiating cells within a certain radius ρ from a differentiated cell. D) Average number of newly differentiated cells exposed to AM within a radius of ρ  = 200 μm from each previously differentiated cell normalized by the amount E(*n*) expected from random placement of all newly differentiated cells in the whole chamber. *N* indicates the total number of newly differentiated cells detected in the present image. The blue error bars indicate the standard deviation of the number of newly differentiated cells within the radii of the previously differentiated cells, while the red error bars illustrate the average counting error due to finite statistics.

The ability to differentiate in AM under perfusion increased with cell density, which was predicted from the results of the numerical simulations of cell-released factor concentrations (Figure 1). Furthermore, differentiation in the perfusion system was homogeneous in the entire chamber at all cell densities (Figure 2c), contrasting the dispersed islands of differentiated cells usually found in batch cultures. However, in general the degree of differentiation was much higher at perfusion with the low concentration adipogenic medium compared to the normal used concentrations (Figure 2a). In agreement with this, perfusion with higher than normal concentrations of adipogenic factors resulted in even less degree of differentiation (data not shown). Further perfusion experiments were therefore performed with the four times lower than normal concentrations of adipogenic factors and serum and throughout the paper referred to as AM and CM (Figure 3, 4, 5, and 6 and Figure S3, S4, S5 and S6). It should be noted that no spontaneous differentiation was observed by perfusion with normal growth medium (GM) (Figure S2b). Close-up images of the morphology of differentiating ASCs in microfluidic perfusion conditions are shown in Figure S2a.

Analyzing time lapse data of differentiating cells, showed that CM had a positive effect on total fat accumulation in the chamber from day 6–10 (Figure 3a-c and Figure S3a-c). Splitting up the analysis of fat content in the chamber into fat vacuole area per cell and fraction of differentiated cells gave additional information about when the released factor act in the differentiation process. As observed for total fat content in the chamber, CM generally had a positive effect on the fraction of cells that differentiated (Figure 3g-i and Figure S3g-i) as well as the average size of the fat vacuole area per cell (Figure 3d-f and Figure S3d-f). The positive effects of CM on the fraction of differentiated cells were recorded already from day 4–8. The differences between CM and AM were highest at low cell density, while less distinguished at the highest cell density (Figure 3a-i and Figure S3a-i). The maximum fraction of cells that differentiated in CM was almost 60% and 40% in AM (Figure 3g-i). The kinetics curve of average fat vacuole area per cell for cells treated with CM displayed a maximum of 800–1000 μm^2^/cell after 12–14 days of differentiation (Figure 3e-f and Figure S3e-f), which is reasonable since a cell can easily be 2500 μm^2^ (50×50 μm, Figure 2 and Figure S2). The decline in average lipid vacuole area per cell found after day 12–14 coincides with the appearance of very large but fewer vacuoles, probably due to vacuolar fusion (Figure S2a, day 21). The results indicate that CM (the auto/paracrine factor) acts early in the adipogenic differentiation process and initiates the differentiation process faster than AM.

To verify the observed effects of CM over AM on adipogenic differentiation, cells were extracted from the respective chambers after 21 days of differentiation and analyzed by reverse transcription real time PCR for expression of key makers in adipocyte development (C/EBPβ, C/EBPα, PPARγ, LPL and adiponectin). All expression markers showed 2–3 fold higher expression in cells treated with CM compared to cells treated with AM (Figure 4a-e), which in addition correlates with the observed increases in lipid accumulation of cells exposed to CM (Figure 2a and 3a-c). Furthermore, expression levels of all markers except *CEBPB* were lower in cell cultures with low cell density than with higher density despite normalization to *ACTB* expression. As a control of adipogenic marker expression, the expression of the late stage markers *ADIPOQ* and *LPL* in ASCs was nearly undetectable while adipocytes showed expression that was several orders of magnitude higher. However, *CEBPB* was expressed only 3–7 fold higher in adipocytes compared to ASCs. Altogether, these results further support the hypothesis that ASCs or adipocytes are releasing an auto/paracrine factor, which has positive effect on adipogenic differentiation.

### Effect of the secreted factor on early phases of differentiation

An additional set of experiments were performed to further pinpoint the effects of the secreted factor. Cells perfused with AM or CM were extracted from the respective chambers after 12 hours and 1, 2, 3, 4, 6 and 8 days of differentiation and analyzed by reverse transcription real time PCR for expression of *CEBPB* and *CEBPD* (early differentiation markers), *PPARG* and *CEBPA* (middle stage markers) and *LPL* and *ADIPOQ* (late stage markers). Treating cells with AM or CM resulted in an expression pattern consistent with the three-level transcriptional cascade [3], [25] (Figure 5 and Figures S4-S5), where expression of *CEBPB* and *CEBPD* precede expression of *PPARG* and *CEBPA* that in turn showed expression before LPL and ADIPOQ. For all markers, CM treatment of cells resulted in higher marker expression than treatment with AM (Figure 5 and Figures S4–S5). Already after 12 hours (earliest time-point measured) CM displayed its effect on early markers. The positive effect of CM was constant or declining compared to AM with time for the early markers. For middle stage markers a significant positive effect of CM was detected day 1 after differentiation initiation, while significant positive effect of CM for late stage markers was detected 3–4 days after differentiation initiation. Furthermore, measurable lipid accumulation was detected about one day after expression of late stage markers. As a control, cells were treated with growth medium (GM) for 8 days or treated with a 1∶1 mixture of GM and conditioned medium from cells cultured in GM in batch cultures. Under these control conditions, expressions of the adipogenic markers *PPARG, CEBPA,* LPL and *ADIPOQ* were almost undetectable (data not shown). Accordingly, neither GM nor the 1∶1 mixture of GM and conditioned medium from cells cultured in GM in batch cultures resulted in visible differentiation the first eight days (data not shown). Thus, these results confirm that the auto/paracrine factor acts very early in the differentiation process (at least before 12 hours after differentiation initiation) upstream of C/EBPβ and C/EBPδ in the transcriptional cascade, and that the auto/paracrine factor is released in response to AM. The experimental design of this study does not allow us to conclude whether the auto/paracrine factor can have pro-adipogenic effect during later stages of differentiation as well.

### Source of factor secretion

The population of cells in the chamber is a mixture of cells at different differentiation stages. It is thus not clear if the ASCs or the mature adipocytes are the source of the secreted factor. The source of the secreted molecule was investigated by two independent analysis. In the first analysis (results shown in Figure 6a and 6b for an experiment exposed to AM at flow rate Q = 500 nL/min at cell density of 271 mm^−2^ ≈ a cell loading suspension of 4×10^5^ cells/mL) we plotted the histograms of the distance between newly differentiated cells and their nearest previously differentiated cell (blue bars), and compared to the histogram of the distance between any cell (differentiated or not) and its nearest neighbor (also independent of whether this neighbor is differentiated) given by the red line. If an already differentiated cell secretes the factor at a higher rate than ASCs, we would expect the undifferentiated nearest neighbor cell to an already differentiated cell to differentiate with a higher probability than the background population, and we would consequently expect the distribution of distances between differentiated cells to be similar to the distribution of nearest neighbor distances between any cell (differentiated or not). However, the distribution of these nearest distances between newly and previously differentiated cells given by the blue bars in the figure is skewed towards larger distances than the distribution of nearest neighbor distances of all cells (red line) (Figure 6b). This indicates that newly differentiated cells are not the nearest neighbors to already differentiated cells, and therefore suggests that differentiated cells do not secrete the factor at a (significantly) higher rate than ASCs. The same results were obtained when analyzing cultures perfused with CM (Figure S6), and the results were furthermore independent of flow rate (data not shown). To ensure the validity of this first analysis we conducted a second independent analysis by at each time step counting the number of newly differentiated cells within a distance of 200 μm from each previously differentiated cell and comparing this number to the expectance from random placement (Figure 6c). Since the occurrence of newly differentiated cells in these regions did not exceed that expected from random placement (Figure 6d), this second analysis reached the same conclusion as the first. Thus, the results indicate that ASCs and cells undergoing differentiation both secrete the factor.

## Discussion

Using microfluidics, it was possible to investigate the importance of auto/paracrine signaling in ASC differentiation into adipocytes without changing other parameters such as direct physical cell-to-cell contact. By controlling cell density and perfusion rate it was possible to almost entirely suppress differentiation at low cell densities in adipogenic differentiation medium while restoring differentiation by perfusion with conditioned medium. Numerical simulations of concentrations of a cell secreted factor predicted that the cells were able to modulate their own near-cell chemical environment at high cell densities, which is consistent with the observed increase in differentiation at higher cell densities. However, even though the cells were able to modulate their own environment at high cell densities, perfusion with CM increased the degree of differentiation even further (2–3 fold). Moreover, the uniform pattern of differentiated cells in the perfusion system compared to the islands of differentiated cells normally observed in batch cultures further points towards effects of auto/paracrine signaling as a cell released factor is spread by convective mass transport throughout the chamber and thereby hindering local high concentrations in the perfusion system. Overall, large variations in degree of differentiation were observed between independent experiments; however, the same trend of the positive effect of CM was seen in all experiments. The observed variations in degree of differentiation were most probably caused by differences in the actual cell seeding density due to difficulties in cell loading using the microfluidic system.

A general aspect connected to the use of conditioned medium is the unknown composition of the medium. The conditioned medium part in CM is supernatant collected from differentiating cells in batch cultures in adipogenic medium at a higher concentration of serum and other adipogenic stimuli compared to the AM used in the perfusion experiments. It is, however, expected that the concentrations in the collected supernatant are lower than the initial concentrations due to degradation and consumption of adipogenic stimuli and other serum components. Although, the concentration might be higher in CM compared to AM, our results indicate that this is not the reason to the increased differentiation in cultures perfused with CM. In fact, as shown in figure 2a, perfusion with AM and CM based on higher concentrations (denoted normal conc. in the figure) of adipogenic factors and serum resulted in much lower differentiation compared to four times lower concentrations (denoted low conc.). Furthermore, because the same source of serum has been used in all tested conditions any variation compared to other serum sources should be eliminated.

The use of indomethacin as adipogenic factor in AM might affect paracrine signaling through prostaglandins, because indomethacin inhibits synthesis of some prostaglandins [41], [42]. Prostaglandins have been shown to have both positive and negative effects on adipogenesis [41]–[43]. However, indomethacin is present in both tested conditions (perfusion with AM or CM). So, even though indomethacin may inhibit synthesis and thereby secretion of some prostaglandins, we still observe a positive effect on adipogenesis of an auto/paracrine factor in CM. Thus altogether, the results indicate that an auto/paracrine factor is necessary for efficient differentiation of ASCs into adipocytes.

Although adipogenic differentiation has been addressed in detail earlier [1], [3], auto/paracrine signaling *within* the human ASC population has to our knowledge not previously been implicated in the adipogenic differentiation process. Other studies have indicated that ASCs or MSCs can indeed be responsive to paracrine factors as both pre-co-culture of human ASCs with human adipocytes [32] and culture of mouse MSCs in conditioned medium collected from either mouse adipose tissue culture [33] or from differentiating osteoblasts [44] have a positive effect on differentiation. In contrast to these results, we present data indicating that human ASCs release an auto/paracrine factor themselves, possibly as a first response to adipogenic stimuli provided in AM, and that the action is early in the differentiation process.

Other studies have indicated that physical cell-to-cell contact causes the increased differentiation at higher cell densities. By the use of pattern clusters with different number of rat marrow MSCs, it has been shown that a small cell size and clusters with an increasing number of cells enhance the adipogenic differentiation, which is partly due to direct physical cell-to-cell contact [34], [35]. It was, however, assumed that no paracrine signaling takes place within a cluster of 5–10 cells. McBeath *et al*. [36], proposed that cell density and thereby cell shape and Rho activity were a determinant of human MSC commitment to either the adipogenic or osteogenic differentiation linage. By patterns of extra cellular matrix islands of different sizes, they showed that a small round cell promoted adipogenesis, while a large spread cell promoted osteogenesis in dual adipogenic/osteogenic medium. As in [34], [35], auto/paracrine effects are presumed not to take place between islands of single cells. However, the fact that a secreted auto/paracrine factor is necessary for differentiation does not rule out that a direct physical contact is also important.

**Figure 7:**
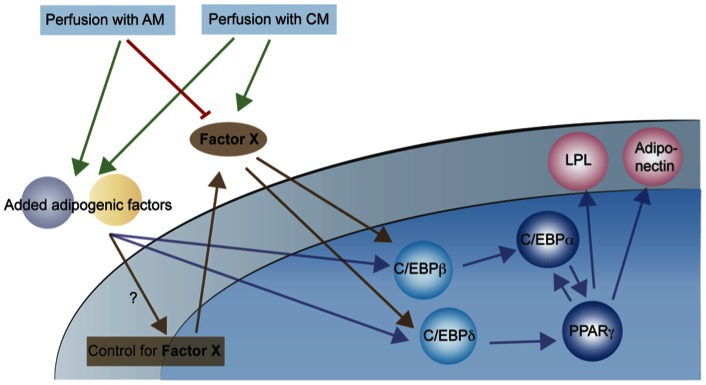
Graphical model of signaling in ASC adipogenic differentiation. ASCs exposed to adipogenic stimuli in AM secrete an unknown factor acting as a positive regulating signal in the differentiation process. This positive cell-released signaling factor is removed during perfusion conditions with AM thereby suppressing differentiation. Differentiation can be restored by perfusion with the factor contained in CM. The auto/paracrine signaling factor is acting in the early phase of differentiation upstream of the transcription factors C/EBPβ and C/EBPδ.

The results indicate that CM has effect early in the differentiation process upstream of C/EBPβ and C/EBPδ in the transcriptional cascade, suggesting involvement in the recruitment phase of adipogenic differentiation (Figure 7). This is consistent with both members of the TGFβ superfamily and WNT ligands which are known to be involved in the adipogenic commitment for both MSCs and preadipocytes [1], [9]–[11]. Therefore, is the component in CM affecting the WNT or TGFβ superfamily pathways or is it an altogether novel pathway? Firstly, modulation of the WNT pathway by CM is not likely a mechanism as removal of WNT ligands by perfusion with AM is not sufficient to induce differentiation in low cell density cultures. Thus, CM contains a positively acting factor, which cannot be involved in relieving suppression based on a solute, as is the case with WNT signaling in adipogenic differentiation. Secondly, CM has effect very early in the differentiation program, as already 12 h after treatment (or earlier) a higher expression of early adipogenic markers (C/EBPβ and C/EBP/δ) is observed in cells perfused with CM compared to cells perfused with AM. It is possible that the secreted factor is BMP4 as exposure of human ASCs and human preadipocytes to BMP4 *both before and throughout* differentiation enhances adipogenesis [12], [13]. Furthermore, *BMP4* mRNA is induced during human adipogenesis and undifferentiated cells rather than adipocytes seems to be the target cells of BMP4 [12]. However, in mouse C3H10T1/2 multipotent cells *pretreatment* with BMP4 followed by induction of differentiation increases adipogenesis [14]. Notably, the effect on differentiation of treatment *both before and throughout* differentiation or *simultaneously* induction of differentiation with BMP4 and normal differentiation medium was not described [14]. Alternatively, others have suggested that a positive acting factor released from mouse fat tissue, and therefore presumably from mature adipocytes, is 3–5 kDa [33] and hence much smaller than BMP-4.

### Conclusion

Based on our results from perfusion cell cultures and following real time PCR analysis, we propose a modified adipogenic differentiation model of human ASCs (MSCs) (Figure 7). Human ASCs exposed to adipogenic stimuli in AM secrete an unknown factor acting as a positive regulating signal in the differentiation process. This positive cell-released signaling factor is removed during perfusion conditions with AM thereby suppressing differentiation. Differentiation can, however, be restored by perfusion with the factor contained in CM. The auto/paracrine signaling factor is acting at least in the early phase of differentiation upstream of the transcription factors C/EBPβ and C/EBPδ. Further studies will reveal the identity of the auto/paracrine factor and the experimental design including the microfluidic systems employed in this study is very suitable for testing of possible candidates.

## Materials and Methods

### Microfluidics and chips

Construction, fabrication and characterization of the employed microfluidic systems have been described elsewhere [39], [40]. The employed microfluidic cell culture chips were made of poly(methyl methacrylate) (PMMA) (Plexiglas XT 20070, Röhm GmbH, Germany and Solaris Clear S000, PSC A/S, Denmark) and fabricated by micro milling followed by a UV assisted local heat bonding [40]. In short, the individual layers of the chip were cleaned with 70% EtOH or isopropanol before being exposed to UV (DYMAX, 5000 EC with bulb 36970, CT, USA) for 90 s. Following UV exposure the layers were sandwiched between two glass slides in an alignment setup and bonded for 30 min in a laboratory press (PW 10 H, P/O/Weber, Germany) pre-heated to 90°C and at an initial applied pressure of approximately 8 kN for chips measuring 26 mm (*w*) ×76 mm (*l*) and 12.50 kN for chips measuring 52 mm (*w*) ×76 mm (*l*). The chips measuring 26 mm (*w*) ×76 mm (*l*) had a total thickness of 3.5 mm and the chips measuring 52 mm (*w*) ×76 mm (*l*) had a total thickness of 3.0 mm. Depending on the specific design, they were composed of individual sheets of PMMA ranging from 0.5 mm to 2 mm in thickness. The bottom layer of all chips was 0.5 mm for reduced optical path length from the sample to the objective. Inlets are spaced 2.25 mm and placed along each chip side in groups of 8 to interface pumps and cell loading chips. The cell culture chambers had a footprint of 1.5 mm (*w*) ×6 mm (*l*) capped by isosceles triangles. Chamber height was 500 µm. For the chips measuring 26 mm (*w*) ×76 mm (*l*) the inlet and outlet channels were connected at the top surface of the chamber. For the chips measuring 52 mm (*w*) ×76 mm (*l*), the inlet were connected to the top of the chamber and the outlet to the bottom of the chamber. The chips contained from eight to twenty-four separate cell culture chambers. Each of the cell culture chambers was individually addressed. The micropumps addressing the chip were controlled with either a custom made computer controller [40] or a LEGO® Mindstorm controller [39]. All flow rates described below are average flow rates.

### Cells and cell culture medium

Human adipose stem cells (ASCs) were isolated from the stromal vascular fraction from three female donors collected after written informed consent according to Southern Norway Regional Ethic Committee REK-SØR approval number REK-6037a. Cells were expanded as a pool from these donors as described earlier [7], for 5–7 passages and then stored in liquid nitrogen and thawed for further culture as described here. Cells were cultured in growth medium (GM) consisting of DMEM/F-12+GlutaMax™ (31331, GIBCO) supplemented with 15% v/v newborn calf serum (NCS; N4762, Sigma), 100 U/mL penicillin, and 100 μg/mL streptomycin (P4333, Sigma). Of note, expanding and differentiating ASCs in medium containing fetal or newborn calf serum did not in our hands result in noticeable differences (data not shown), thus NCS was used in subsequent cultures for reasons of availability. Upon conventional static cell culturing, cells were incubated at 37°C and 5% CO_2_.

Adipogenic differentiation in standard batch cultures was performed in GM supplemented with 15% v/v NCS, 0.5 mM IBMX (isobutyl-methylxanthine) (I5879, Sigma), 1 µM dexamethasone (D4902, Sigma), 0.2 mM indomethacin (I7378, Sigma), and 10 µg/mL insulin (I9278, Sigma) as described earlier [8]. Unless mentioned otherwise, for adipogenic differentiation in the microfluidic perfusion systems, concentrations of serum and adipogenic factors used in what we referred to as the adipogenic medium (AM) were 4 times lower than in the batch cultures (3.75% NCS, 0.125 mM IBMX, 0.25 µM dexamethasone, 0.05 mM indomethacin and 2.5 µg/mL insulin. Of note, these lower concentrations were found in initial experiments to result in a much higher proportion of differentiated cells than those used for differentiation in batch cultures (Figure 2a).

Conditioned medium (CM) was a 1∶1 mixture of supernatant from ASCs undergoing differentiation in batch cultures and fresh AM to ensure sufficient supply of nutrients. 1.5 times the concentration of IBMX, dexamethasone, indomethacin and insulin (1.5×AM) was used in the mixture instead of 1×AM to compensate for an expected consumption/degradation of the adipogenic stimuli in the collected supernatant. However, as the collected supernatant still probably contains some insulin, IBMX, indomethacin and dexamethasone, only 1.5×AM was used instead of 2×AM to obtain approximately 1×AM. For production of supernatant from differentiating cells, ASCs were grown to a cell confluence of approximately 80–90%, before the cell culture medium was changed to adipogenic medium as described for batch cultures to induce the differentiation. Half of the conditioned differentiation medium was collected normally at day 4, 8, 12 and 16 after onset of differentiation and stored at 4°C. The collections of conditioned supernatant from the four days during the differentiation period were pooled, aliquoted and stored at −20°C. All differentiation media was freshly prepared just before use.

### Microfluidic cell culture

To avoid contamination of the cell culture by bacteria or fungi, all system preparations and changes of media reservoirs have been performed in a laminar flow bench and by the use of aseptic working procedures. Liquid glass vials, caps, and silicone/poly (tetrafluoroethylene) (PTFE) tubing were sterilized by autoclaving before use. Glass vials or vial chips, tubing and connections to pumps were assembled onto the system base plate. The cell culture chip and tubes connecting the liquid reservoirs to the pumps or vial chips were filled separately with Milli-Q water to remove bubbles, before assembling the cell culture chip to the base plate [39], [40]. Inlet and outlet reservoirs were coupled with PTFE tubing (BOLA 1810-10, Bohlender GmbH, Germany) and supplied with air supplemented with 5% CO_2_ through a sterile filter. To avoid formation of gas bubbles in the microfluidic network a pressure of 0.3 bars was put on the flow system during the whole system preparation and cell culture period, only interrupted when for instance changing liquid or liquid reservoirs.

The assembled microfluidic system was first sterilized by flushing with 0.5 M NaOH for 20 minutes at a flow rate of 5.2 μL/min. The chip was then flushed with sterile water for 30 minutes at a flow rate of 5.2 μL/min to remove all NaOH. For cell adhesion to the surface, the chip was coated by perfusion of 40 μg/mL collagen (C3867, Sigma) in sterile water at a flow rate of 5.2 μL/min for 15 minutes. The chamber was subsequently perfused for 45 minutes at 250 nL/min with the collagen solution. During coating the system was placed in an incubator at 37°C. The cell culture chip was then flushed with cell culture medium for 30 minutes at a flow rate of 5.2 μL/min or 250 nL/min overnight to remove excess collagen. ASCs were resuspended in cell culture medium added 60% v/v NCS to increase the viscosity of the suspension and thereby improve the uniformity of the cell loading. 10 µL cell suspension was, after removal of tubings, loaded into the embedded wells in the cell loading chip [40]. Various cell suspension densities have been applied (see figure captions). Cells were introduced into the cell culture chambers by setting the pumps to run backwards at high speed to enable uniform cell loading. For the LEGO® motors a LEGO® Mindstorms flow program of 10 rotations in 6 sec (∼ flow rate of 65 µL/min) was employed**.** After cell loading, the outlet tubings were attached to the cell loading chip again under aseptic conditions. The system was then placed in an incubator at 37°C and 5% CO_2_. The cells were perfused with a low flow rate of 33 nL/min for 4 h to allow cell attachment. Following this attachment phase, the flow was change to the given cell culture perfusion rate. Changes of cell culture medium or supply of fresh medium has been performed either by changing the glass vials or by suction of remaining medium in the vial chips followed by refilling of the reservoirs. Cell culture medium was changed at least every 4 days.

### Adipogenic differentiation in standard batch cell culture

At a cell confluence of approximately 80–90%, the cell culture medium was changed to adipogenic differentiation medium to induce the differentiation. The differentiation was continued up to three weeks with normally half of the differentiation medium changed every 3–4 days.

### Adipogenic differentiation in perfusion cell culture

Differentiation was induced at a cell confluence of approximately 80–90%. When testing differentiation at different cell densities, the differentiation was induced the day after cell loading. The cells were perfused with the different tested differentiation media and different tested flow rates. Medium reservoirs were exchanged with fresh medium at least every 4 days. For each differentiation experiment, one chamber was grown in normal cell culture medium as a negative control.

### Gene expression analysis

Total cellular RNA was purified by using the RNeasy Micro kit (Qiagen, 74004). Cells were lysed directly in the chamber using the lysis buffer provided in the Qiagen RNeasy Micro kit. The lysis was collected in microtubes and purified according to manufacturer's instructions (Qiagen, 12/2007). The RNA was converted to cDNA using the High Capacity cDNA Reverse Transcription Kit (Applied Biosystems, 4374966) according to the manufacturer's instructions (06/2010). Real time PCR was conducted using the TaqMan® Gene Expression Assays from Applied Biosystems. The cDNA from one chamber was split into seven tubes and mixed with the specific TaqMan® Gene Expression Assay (Applied Biosystems 4331182, *ACTB* (β-actin) ID: Hs99999903_m1, *CEBPA* (C/EBPα) ID: Hs00269972_s1, *CEBPB* (C/EBPβ) ID: Hs00270923_s1, *CEBPD* (C/EBPδ) ID: Hs00270931_s1, *PPARG* (PPARγ) ID: Hs01115513_m1, *LPL* (LPL) ID: Hs00173425_m1 and *ADIPOQ* (adiponectin) ID: Hs00605917_m1), TaqMan® Gene Expression Master mix (Applied Biosystems, 4370048) and RNase-free water according to the manufacturer's instructions (Applied Biosystems 11/2010). The respective C_t_ values obtained after analysis in a Chroma4 real time PCR machine (MJ Research, the program run at 50°C for 2 minutes, 95°C for 10 minutes and 40 cycles of 15 sec at 95°C and 1 minute at 60°C) were normalized to the C_t_ value of β-actin (e.g. C_t_(C/EBPα)-C_t_(β-actin). β-actin has been shown to be a good candidate to use for normalization since it does not change significantly over time [45].

### Imaging and image analysis of ASC differentiation

Phase contrast images of ASC differentiation were normally acquired every second day by a Zeiss Axio Observer.Z1 microscope equipped with a 10x/0.3 Plan-Neofluar objective, and a Zeiss Axiocam MRm B/W camera. A scan of each cell culture chamber, all images acquired with a z-stack of 5 slices (6 µm between each slice), were recorded with an exposure time of 5 msec. The images were processed by applying the AxioVision Extended Focus module on the z-stacks to obtain the best focused image, stitching the individual images together and finally converting the stitched images to one image.

Differentiation was quantified by two different methods. In the first method, differentiation was estimated as ability of cells to sequester lipid droplets. The images were analyzed in ImageJ. The area of lipid-filled droplets was measured by summing all areas with at least 2 pixels in diameter, where each pixel had a gray value between 31347 and 65520. The total cell area at start of differentiation was measured by marking pixels with gray values of 12076 or more. Dividing the area of the lipid droplets with the total cell area at the start of the experiment is expressed as lipid accumulation in relative units (r.u.).

In the second method, we automatically detected the number of differentiated cells from the images at each time step using custom Matlab software by clustering neighboring vacuoles belonging to the same cell and obtained the fraction of differentiated cells by normalizing with a manual count of all cells at the first time point. Following intensity-adjustment (using Matlab's built-in contrast-limited adaptive histogram equalization function adapthisteq), individual vacuoles were detected by thresholding (using a relative intensity of *I*_rel_ = *I*/*I*_max_ = 0.9 as threshold). Regions satisfying the following criteria were accepted: (i) region contains at least 2 pixel, (ii) region contains at most 250 pixel (large single vacuoles were no larger than ∼100 pixel), (iii) region major axis to minor axis did not exceed 3 (violation signifies a severely aspherical object), and (iv) intensity gradient towards the edges of corresponding region in original image. This last criterion was implemented by requiring the mean relative intensity gradient away from the region centroid 
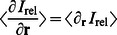
 be negative, where the gradient was approximated numerically for a detected region centered in (*x*_n_,*y*_n_) as
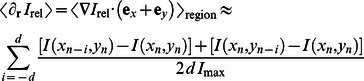
where *d* is the distance in units of pixel over which the gradient is approximated, which was taken as the aforementioned minor axis of each individual region, and **e** *_x_*,**e** *_y_* are the unit vectors in the two coordinate directions of the image. The accuracy of this method was determined to exceed 99% in two independent cases. Segmentation (clustering) of detected vacuoles from the same cell was achieved by water shedding, using markers of each differentiated cell extracted from the intensity-adjusted image by twice convoluting with Gaussian kernels of different variance to smooth borders between neighbouring vacuoles and subsequently detecting and dilating (using a circular structuring element of 10 pixel radius) subregions of local minima within each vacuolar region. Detected vacuoles were then assigned to the correct regions (differentiated cells), and differentiated cells were linked in time based on the metric distance between subsequent images requiring that relative area changes did not exceed 50% (requirements were relaxed for regions containing up to 50 pixel since initial large growth was typically observed), working iteratively to ensure that the overall distance between all linked clusters is minimized between the two frames. Details of this second image analysis procedure and its implementation can be found in [46].

### Simulations

To simulate the distribution of secreted factor in the microfluidic system and well plates, we solved numerically the governing time-dependent advection-diffusion equation for the concentration *c*(**x**,*t*) of this factor
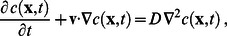
as a function of space **x** and time *t* using the commercial finite element software COMSOL Multiphysics version 3.5a. In the above equation, **v** is the velocity vector and *D* is the molecular diffusivity of the factor. We solved the equation in a two-dimensional domain – corresponding to the transverse middle of the channel – using the analytical solution to the velocity field obtained from solution of the steady-state (time-independent) Stokes equation [47]. We used a flux boundary condition at the layer of the cells to simulate the factor secretion, assuming that each cell secretes continuously everywhere from its surface and at the same rate,



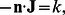
where **n** is the outward-pointing normal vector and **J** is the flux of factor concentration. Since each cell releases 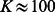 molecules per second [48], [49], the number *k* was estimated as



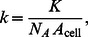
where 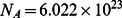 mol^−1^ is Avogadro's number and *A*_cell_ is the area associated with each cell. To simulate high and low density experiments we varied *A*_cell_ from π×(10 μm)^2^  = 314 μm^2^ (corresponding to complete confluence with cells of 10 μm radius) to 2513 μm^2^ (corresponding to an average cell-cell distance of ∼5.6 cell radii (10 μm radius) similar to our low-density experiments) and consequently used 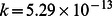 mol m^−2^ s^−1^ and 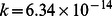
mol m^−2^ s^−1^. Finally, we used no-flux boundary conditions on all other walls as well as the open surface of the well plate. For simulation of the microfluidic experiments we furthermore used zero concentration at the inlet (placed sufficiently downstream that it does not influence the secretion) and convective outflow at the outlet.

## Supporting Information

Figure S1 **Microfluidic system.** The system consists of eight-channel peristaltic pumps, chip for culturing cells, and various containers for storing of liquid. These components are connected to each other via tubes made entirely of PDMS or by Teflon tubing. A) Schematic view of the fluidic path in the microfluidic system. The diagram shows the fluidic path of one of the 8–24 parallel flow paths (depending on chip used). B) Example of a microfluidic system with LEGO® motors and controllers for fluidic actuation driving a 16 chamber chip (insert). A pressure of 0.3 bar of atmospheric air, added 5% CO_2_, was applied to the whole microfluidic network to avoid formation of bubbles. Orange arrows indicate the fluidic path and blue arrows the air pressure path.
(TIF)

Figure S2 **Differentiation of ASCs into adipocytes in adipogenic differentiation medium at perfusion cell culture conditions.** ASCs were induced to differentiate at a flow rate of 500 nL/min ∼ exchange of the entire medium in the cell culture chamber every 10 minutes. A) Imaging after 6, 12, 16 and 21 days of differentiation. The cells were able to differentiate and accumulate fat as shown by the lipid-filled droplets indicated by arrows. B) ASCs after 21 days of culture in normal growth medium at a flow rate of 500 nL/min as a negative control. C) Differentiation of ASCs in static cell culture conditions after 21 days of differentiation as a reference.
(TIF)

Figure S3 **Time course study of lipid accumulation during adipogenic differentiation.** Experiment II out of two independent experiments was followed over time. An image of cells in the entire cell culture chamber was captured every second day and relative lipid accumulation in relative units (A–C), lipid area per cell (D–F) and fraction of differentiated cells (G–I) was determined as described in material and methods. Corresponding results from experiment I are shown in Figure 3. Each graph in the diagrams corresponds to analysis of one chamber.
(TIF)

Figure S4 **Effect of conditioned medium on gene expression of adipogenic markers early in the differentiation process.** In an additional set of experiments ASCs were loaded at a cell suspension density of 2×10^5^ cells/mL and induced to differentiate at a flow rate of 500 nL/min in AM or CM. Gene expression of the adipogenic markers *CEBPB, CEBPD, CEBPA, PPARG, LPL* and *ADIPOQ* was analyzed by RT-PCR of all cells in one cell culture chamber after 12 hours, 1, 2, 3, and 4 days of differentiation. The results shown are from experiment II out of three independent experiments. Results from experiment I is shown in Figure 5 and experiment III is shown in Figure S5. Relative gene expression to *ACTB* shown on the left y-axis for A) *CEBPB*, B) *CEBPD*, C) *CEBPA*, D) *PPARG*, E) *LPL* and F) *ADIPOQ*. The corresponding lipid accumulation is shown on the right y-axis. Note the y-axis scales denoting the real-time PCR results are different in order to clearly visualize effects of CM on all adipogenic markers. Each point on the graphs corresponds to analysis of cells from one chamber.
(TIF)

Figure S5 **Effect of conditioned medium on gene expression of adipogenic markers early in the differentiation process.** In an additional set of experiments ASCs were loaded at a cell suspension density of 2×10^5^ cells/mL and induced to differentiate at a flow rate of 500 nL/min in AM or CM. Gene expression of the adipogenic markers *CEBPB, CEBPD, CEBPA, PPARG, LPL* and *ADIPOQ* was analyzed by RT-PCR of all cells in one cell culture chamber after 12 hours, 1, 2, 3, 4, 6, and 8 days of differentiation. The results shown are from experiment III out of three independent experiments. Results from experiment I is shown in Figure 5 and experiment II is shown in Figure S4. Relative gene expression to β-actin shown on the left y-axis for A) *CEBPB*, B) *CEBPD*, C) *CEBPA*, D) *PPARG*, E) *LPL* and F) *ADIPOQ*. The corresponding lipid accumulation is shown on the right y-axis. Note the y-axis scales denoting the real-time PCR results are different in order to clearly visualize effects of CM on all adipogenic markers. Each point on the graphs corresponds to analysis of cells from one chamber.
(TIF)

Figure S6 **Source of factor secretion.** A) Distributions of the average distances between newly differentiated cells and the closest already differentiated cell (blue) compared to the average distance between all nearest neighbor cells (red) for data at the density 270 cells/mm^2^ exposed to CM at Q = 500 nL min^−1^. Each plot in each panel is normalized by its largest value. The distributions of distances between newly differentiated cells and previously differentiated cells are skewed towards larger distances than the distribution of nearest neighbor distances. This indicates that the newly differentiated cells are not the nearest neighbors to already differentiated cells, which would be expected if differentiated cells secrete the critical chemical at a higher rate. At day 3 only few differentiated cells exist, and the distribution of distances between already differentiated and newly differentiated cells is much wider than at later times; this illustrates that all cells (both differentiated and undifferentiated) do secrete the differentiation factor. B) Average number of newly differentiated cells within a radius of ρ  = 200 μm from each previously differentiated cell normalized by the amount E(n) expected from random placement of all newly differentiated cells in the whole chamber. N indicates the total number of newly differentiated cells detected in the present image. The blue errorbars indicate the standard deviation of the number of newly differentiated cells within the radii of the previously differentiated cells, while the red errorbars illustrate the average counting error due to finite statistics (see text for details). The average amount of newly differentiated cells within the interrogation regions of previously differentiated cells is in agreement what one would expect from random placement, which suggests that there is no enhanced secretion of the critical factor by differentiated cells.
(TIF)
