# Supplementary figures and images for: The Role of Paracrine and Autocrine Signaling in the Early Phase of Adipogenic Differentiation of Adipose-derived Stem Cells
Source: PLoS One. 2013 May 28;8(5):e63638. doi: 10.1371/journal.pone.0063638 (PMC3665830; doi:10.1371/journal.pone.0063638)

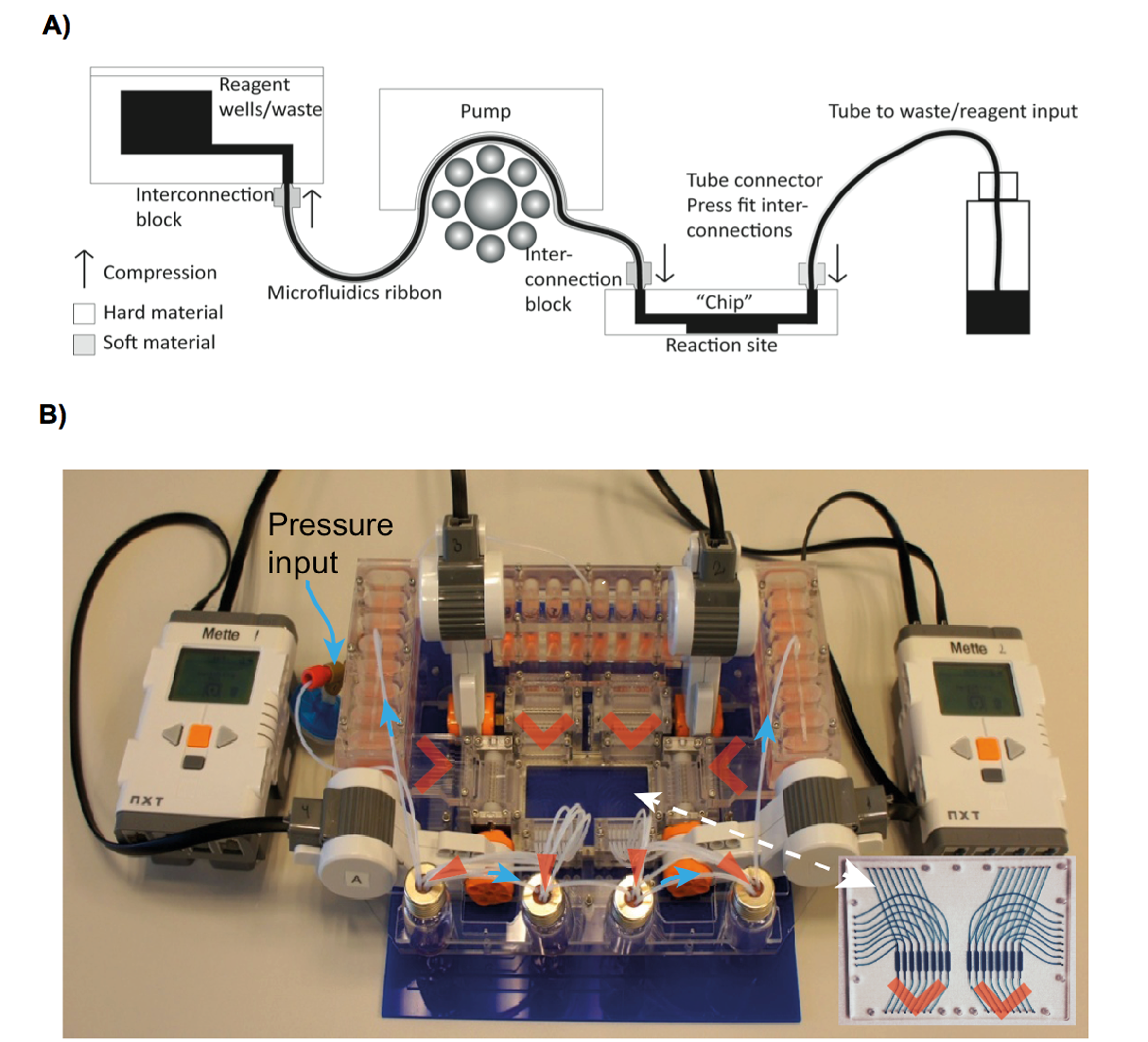

Supplement: Figure S1 — Microfluidic system. The system consists of eight-channel peristaltic pumps, chip for culturing cells, and various containers for storing of liquid. These components are connected to each other via tubes made entirely of PDMS or by Teflon tubing. A) Schematic view of the fluidic path in the microfluidic system. The diagram shows the fluidic path of one of the 8–24 parallel flow paths (depending on chip used). B) Example of a microfluidic system with LEGO® motors and controllers for fluidic actuation driving a 16 chamber chip (insert). A pressure of 0.3 bar of atmospheric air, added 5% CO2, was applied to the whole microfluidic network to avoid formation of bubbles. Orange arrows indicate the fluidic path and blue arrows the air pressure path. (TIF) [file pone.0063638.s001.tif]

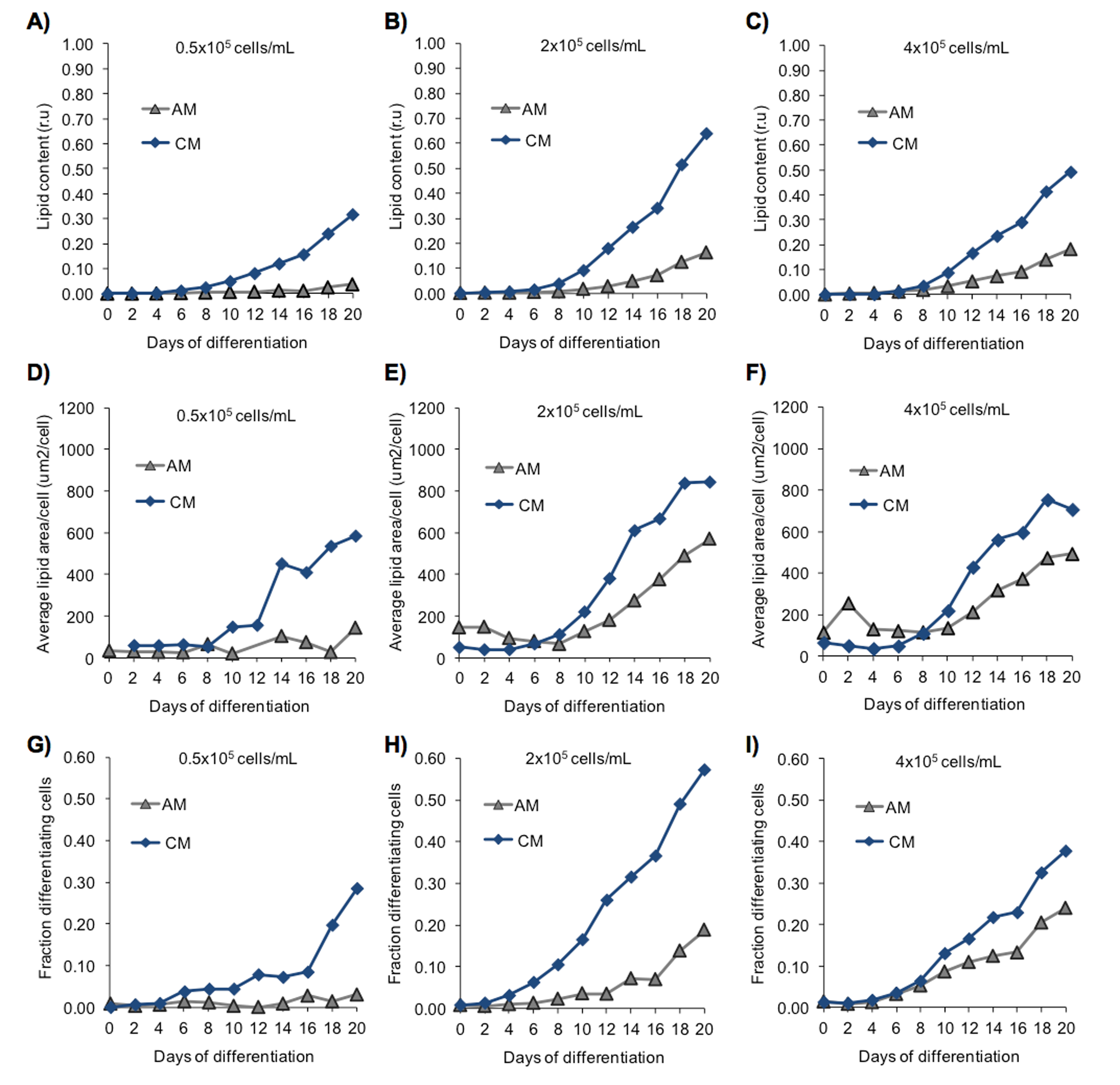

Supplement: Figure S2 — Differentiation of ASCs into adipocytes in adipogenic differentiation medium at perfusion cell culture conditions. ASCs were induced to differentiate at a flow rate of 500 nL/min ∼ exchange of the entire medium in the cell culture chamber every 10 minutes. A) Imaging after 6, 12, 16 and 21 days of differentiation. The cells were able to differentiate and accumulate fat as shown by the lipid-filled droplets indicated by arrows. B) ASCs after 21 days of culture in normal growth medium at a flow rate of 500 nL/min as a negative control. C) Differentiation of ASCs in static cell culture conditions after 21 days of differentiation as a reference. (TIF) [file pone.0063638.s002.tif]

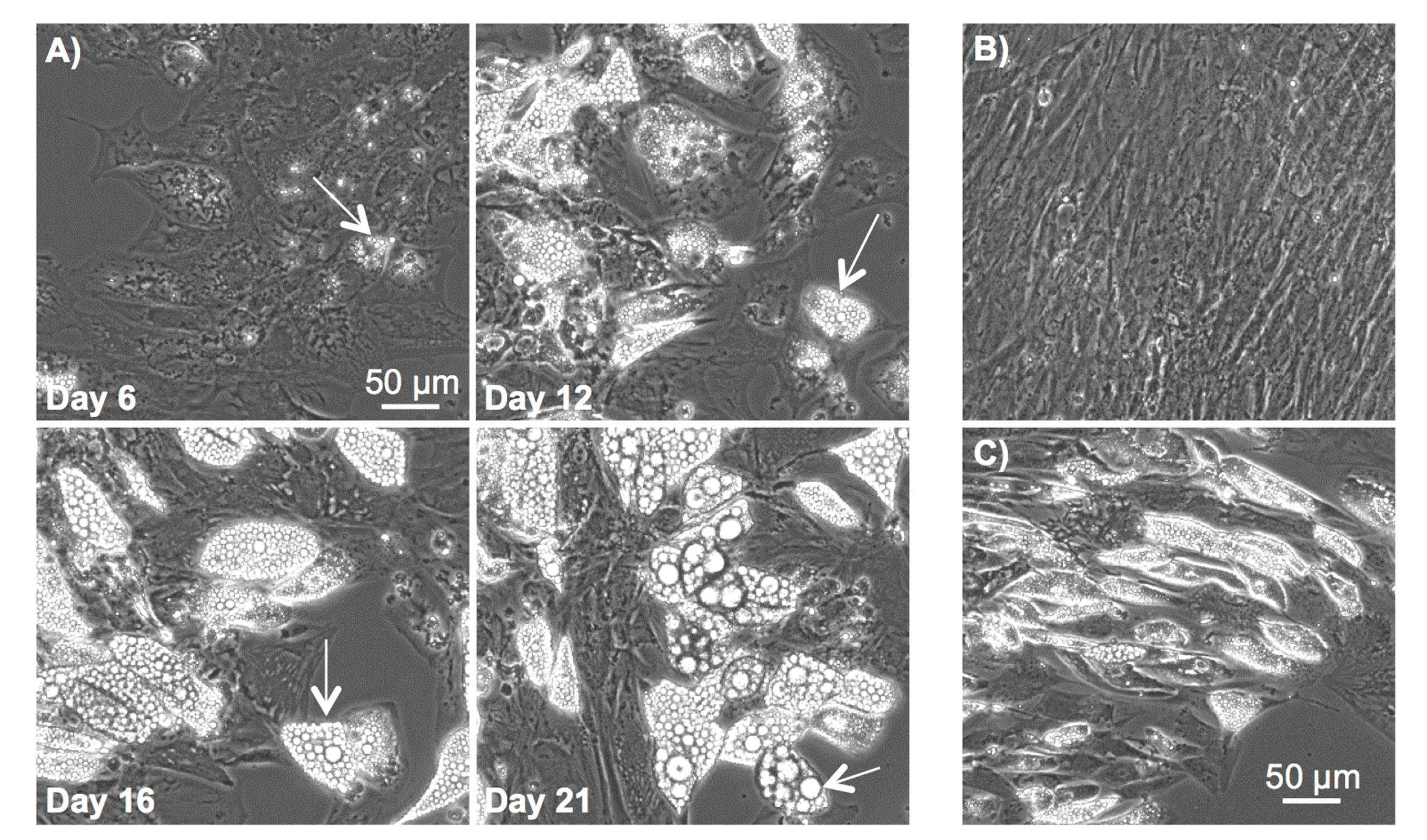

Supplement: Figure S3 — Time course study of lipid accumulation during adipogenic differentiation. Experiment II out of two independent experiments was followed over time. An image of cells in the entire cell culture chamber was captured every second day and relative lipid accumulation in relative units (A–C), lipid area per cell (D–F) and fraction of differentiated cells (G–I) was determined as described in material and methods. Corresponding results from experiment I are shown in Figure 3. Each graph in the diagrams corresponds to analysis of one chamber. (TIF) [file pone.0063638.s003.tif]

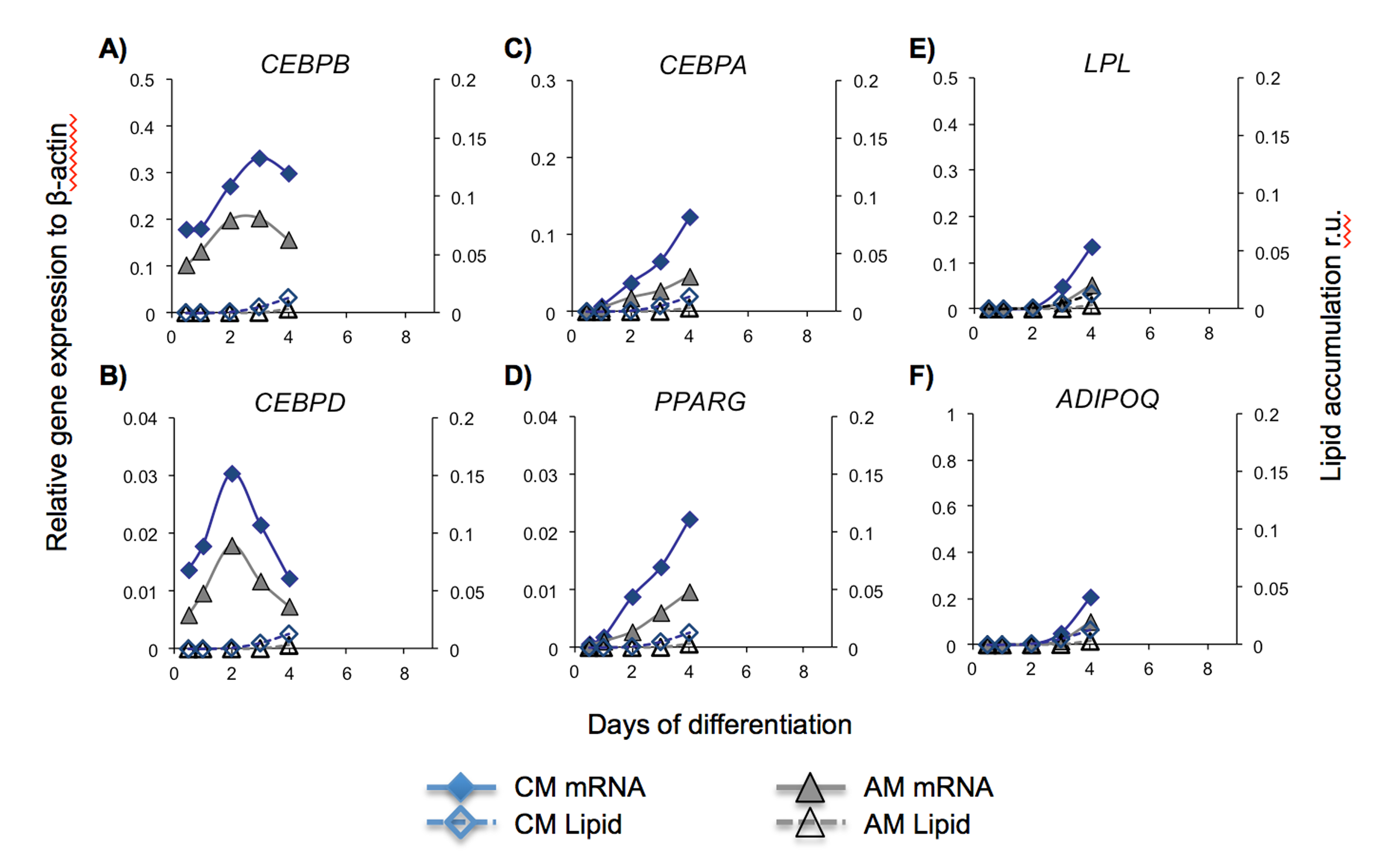

Supplement: Figure S4 — Effect of conditioned medium on gene expression of adipogenic markers early in the differentiation process. In an additional set of experiments ASCs were loaded at a cell suspension density of 2×10^5^ cells/mL and induced to differentiate at a flow rate of 500 nL/min in AM or CM. Gene expression of the adipogenic markers CEBPB, CEBPD, CEBPA, PPARG, LPL and ADIPOQ was analyzed by RT-PCR of all cells in one cell culture chamber after 12 hours, 1, 2, 3, and 4 days of differentiation. The results shown are from experiment II out of three independent experiments. Results from experiment I is shown in Figure 5 and experiment III is shown in Figure S5. Relative gene expression to ACTB shown on the left y-axis for A) CEBPB, B) CEBPD, C) CEBPA, D) PPARG, E) LPL and F) ADIPOQ. The corresponding lipid accumulation is shown on the right y-axis. Note the y-axis scales denoting the real-time PCR results are different in order to clearly visualize effects of CM on all adipogenic markers. Each point on the graphs corresponds to analysis of cells from one chamber. (TIF) [file pone.0063638.s004.tif]

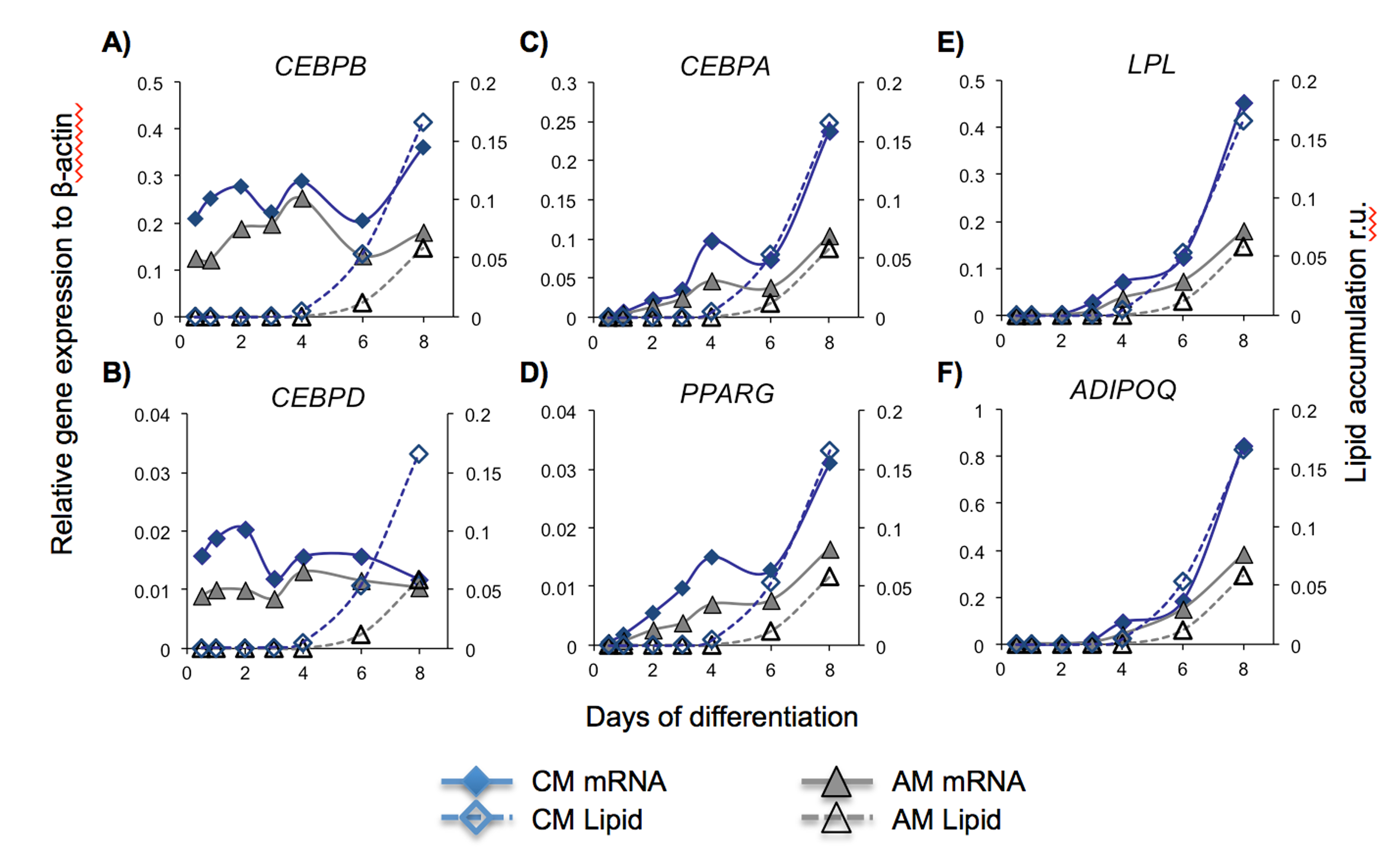

Supplement: Figure S5 — Effect of conditioned medium on gene expression of adipogenic markers early in the differentiation process. In an additional set of experiments ASCs were loaded at a cell suspension density of 2×10^5^ cells/mL and induced to differentiate at a flow rate of 500 nL/min in AM or CM. Gene expression of the adipogenic markers CEBPB, CEBPD, CEBPA, PPARG, LPL and ADIPOQ was analyzed by RT-PCR of all cells in one cell culture chamber after 12 hours, 1, 2, 3, 4, 6, and 8 days of differentiation. The results shown are from experiment III out of three independent experiments. Results from experiment I is shown in Figure 5 and experiment II is shown in Figure S4. Relative gene expression to β-actin shown on the left y-axis for A) CEBPB, B) CEBPD, C) CEBPA, D) PPARG, E) LPL and F) ADIPOQ. The corresponding lipid accumulation is shown on the right y-axis. Note the y-axis scales denoting the real-time PCR results are different in order to clearly visualize effects of CM on all adipogenic markers. Each point on the graphs corresponds to analysis of cells from one chamber. (TIF) [file pone.0063638.s005.tif]

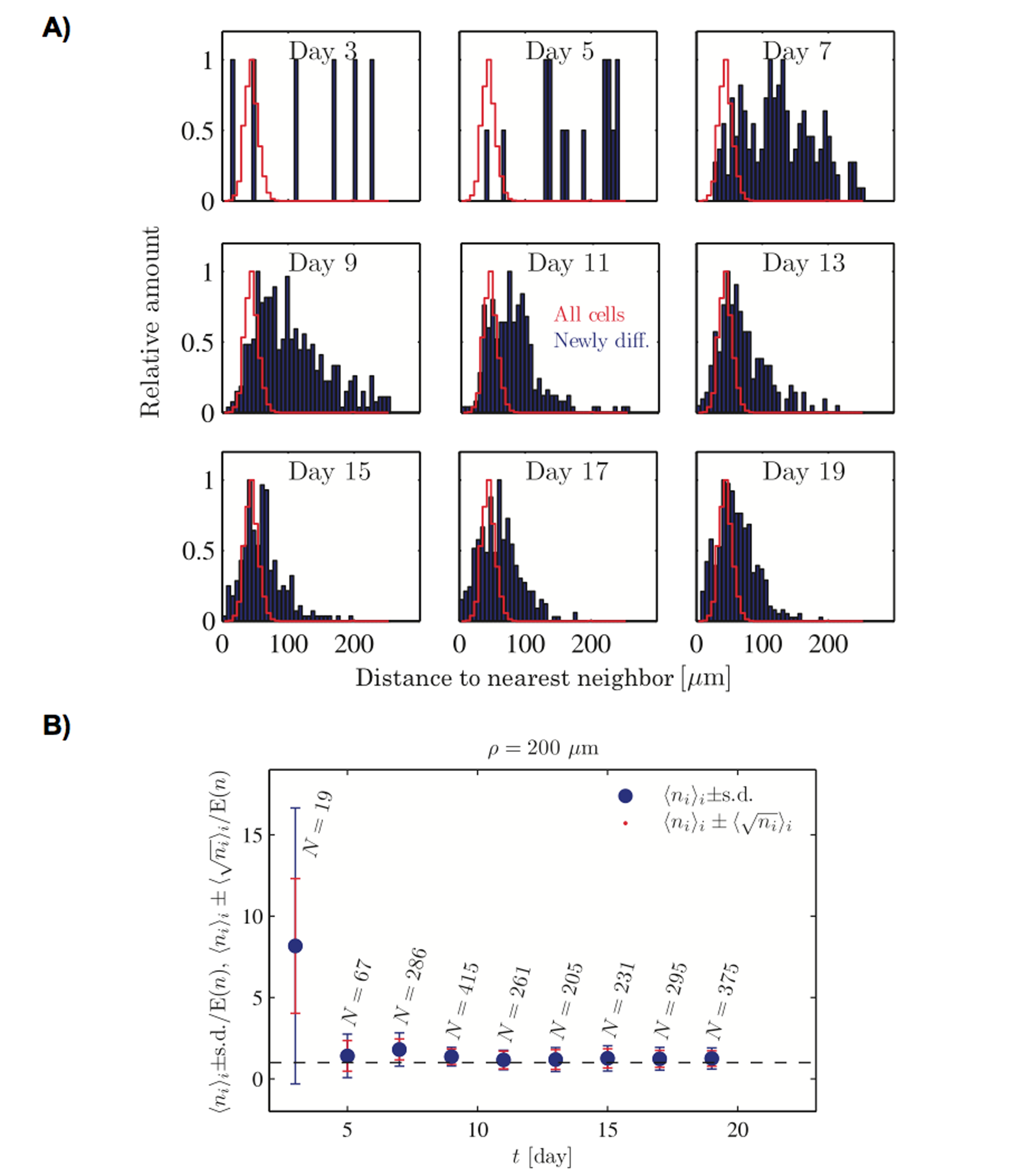

Supplement: Figure S6 — Source of factor secretion. A) Distributions of the average distances between newly differentiated cells and the closest already differentiated cell (blue) compared to the average distance between all nearest neighbor cells (red) for data at the density 270 cells/mm2 exposed to CM at Q = 500 nL min−1. Each plot in each panel is normalized by its largest value. The distributions of distances between newly differentiated cells and previously differentiated cells are skewed towards larger distances than the distribution of nearest neighbor distances. This indicates that the newly differentiated cells are not the nearest neighbors to already differentiated cells, which would be expected if differentiated cells secrete the critical chemical at a higher rate. At day 3 only few differentiated cells exist, and the distribution of distances between already differentiated and newly differentiated cells is much wider than at later times; this illustrates that all cells (both differentiated and undifferentiated) do secrete the differentiation factor. B) Average number of newly differentiated cells within a radius of ρ = 200 μm from each previously differentiated cell normalized by the amount E(n) expected from random placement of all newly differentiated cells in the whole chamber. N indicates the total number of newly differentiated cells detected in the present image. The blue errorbars indicate the standard deviation of the number of newly differentiated cells within the radii of the previously differentiated cells, while the red errorbars illustrate the average counting error due to finite statistics (see text for details). The average amount of newly differentiated cells within the interrogation regions of previously differentiated cells is in agreement what one would expect from random placement, which suggests that there is no enhanced secretion of the critical factor by differentiated cells. (TIF) [file pone.0063638.s006.tif]
